# Adaptation of the protein translational apparatus during ATDC5 chondrogenic differentiation

**DOI:** 10.1016/j.ncrna.2022.02.003

**Published:** 2022-02-20

**Authors:** Mandy M.F. Steinbusch, Guus G.H. van den Akker, Andy Cremers, Adhiambo M.A. Witlox, Heleen M. Staal, Mandy J. Peffers, Lodewijk W. van Rhijn, Marjolein M.J. Caron, Tim J.M. Welting

**Affiliations:** a Laboratory for Experimental Orthopedics, Department of Orthopedic Surgery, Maastricht University, P.O. Box 5800, 6202 AZ, Maastricht, the Netherlands; b Laboratory for Experimental Orthopedics, Department of Orthopedic Surgery, Maastricht University Medical Center, P.O. Box 5800, 6202 AZ, Maastricht, the Netherlands; c Department of Musculoskeletal Biology, Institute of Life Course and Medical Sciences, University of Liverpool, William Henry Duncan Building, 6 West Derby Street, L7 8TX, Liverpool, United Kingdom

**Keywords:** ATDC5, Chondrogenic differentiation, Ribosome, Translation, snoRNA

## Abstract

**Introduction:**

Ribosome biogenesis is integrated with many cellular processes including proliferation, differentiation and oncogenic events. Chondrogenic proliferation and differentiation require a high cellular translational capacity to facilitate cartilaginous extracellular matrix production. We here investigated the expression dynamics of factors involved in ribosome biogenesis during *in vitro* chondrogenic differentiation and determined whether protein translation capacity adapts to different phases of chondrogenic differentiation.

**Materials:**

SnoRNA expression during ATDC5 differentiation was analyzed by RNA sequencing of samples acquired from day 0 (progenitor stage), 7 (chondrogenic stage) and day 14 (hypertrophic stage). RT-qPCR was used to determine expression of fibrillarin, dyskerin, UBF-1, Sox9, Col2a1, Runx2, Col10a1 mRNAs and 18S, 5.8S and 28S rRNAs. Protein expression of fibrillarin, dyskerin and UBF-1 was determined by immunoblotting. Ribosomal RNA content per cell was determined by calculating rRNA RT-qPCR signals relative to DNA content (SYBR Green assay). Total protein translational activity was evaluated with a puromycilation assay and polysome profiling.

**Results:**

As a result of initiation of chondrogenic differentiation (Δt0-t7), 21 snoRNAs were differentially expressed (DE). Hypertrophic differentiation caused DE of 23 snoRNAs (Δt7-t14) and 43 when t0 was compared to t14. DE snoRNAs, amongst others, target nucleotide modifications in the 28S rRNA peptidyl transferase center and the 18S rRNA decoding center. UBF-1, fibrillarin and dyskerin expression increased as function of differentiation and displayed highest fold induction at day 5–6 in differentiation. Ribosomal RNA content per cell was significantly increased at day 7, but not at day 14 in differentiation. Similar dynamics in translational capacity and monosomal ribosome fraction were observed during differentiation.

**Conclusion:**

The expression of a great number of ribosome biogenesis factors is altered during chondrogenic differentiation of ATDC5 cells, which is accompanied by significant changes in cellular translational activity. This elucidation of ribosome biogenesis dynamics in chondrogenic differentiation models enables the further understanding of the role of ribosome biogenesis and activity during chondrocyte cell commitment and their roles in human skeletal development diseases.

## 1 Introduction

Ribosome biogenesis is a central cellular process required for production of ribosome subunits to translate proteins from mRNAs. From a number of genetic diseases, it has become apparent that mutations in genes encoding key components of the ribosome biogenesis machinery interfere with cell function and are causative for developmental disorders and associated malignant conditions. For example, RMRP (snoRNA Component Of Mitochondrial RNA Processing Endoribonuclease) and cartilage hair hypoplasia [1], Dyskerin and Dyskeratosis Congenita [2], TCOF1 (Treacle Ribosome Biogenesis Factor 1) and Treacher-Collins syndrome [3], SBDS (Shwachman-Bodian-Diamond syndrome protein) and Shwachman-Diamond syndrome [4] or components of the mature ribosome RPS14 (Ribosomal Protein S14) and 5q syndrome [5] and RPS19 and Diamond Blackfan Anemia [6]. The molecular dissection of these so-called ribosomopathies [7] has unveiled many unprecedented molecular mechanisms and cell type-specific effects of ribosome biogenesis and functions that dictate the capacity to synthesize proteins and support developmental processes and tissue homeostasis.

The majority of ribosomopathies are associated with disturbances of the development of the skeleton, leading to malformations and dwarfisms [8]. Skeletal development depends on endochondral ossification in the growth plates of the developing skeleton. This complex spatiotemporal cellular process encompasses the chondrocytic commitment of progenitor cells in the resting zone of the growth plate, followed by a proliferative burst of the chondrocytes in the proliferative zone of the growth plate. Proliferative chondrocytes then terminally differentiate into hypertrophic chondrocytes. The extracellular matrix left behind by terminally differentiated hypertrophic chondrocytes is required for mineralization and ossification, enabling longitudinal skeletal development [9].

To accommodate the proliferative burst in the growth plate proliferative zone and the production of the cartilaginous extracellular matrix, a large amount of *de novo* synthesized proteins is needed, while the terminal hypertrophic differentiation calls for a major intra- and extracellular proteomic change. Although a number of genes involved in ribosomopathies and their disease-causing mutations have been identified, a role for the ribosome biogenesis process and protein translation in the field of skeletal development remains largely unexplored.

Expression of small nucleolar RNAs (snoRNAs), which site-specifically guide post-transcriptional modification of ribosomal RNA (rRNA), is dynamically regulated upon murine embryonic stem cell differentiation [10]. In yeast and human cells, these rRNA post-transcriptional modifications are required for tuning ribosome translational fidelity [11,12]. However, many aspects of snoRNA function in development and disease remain to be discovered [13,14]. An exciting recent development is the potential contribution of snoRNA-mediated post-transcriptional rRNA modification to ribosome heterogeneity [13,15]. We have previously shown that the ribosome biogenesis factor RMRP snoRNA is dynamically regulated during *in vitro* chondrogenic differentiation [16]. In addition, we demonstrated differential expression of snoRNAs in murine, equine and human cartilage disease or ageing [[16], [17], [18], [19], [20]].

We expect that chondrogenic differentiation is demanding for the growth plate chondrocyte's protein translation apparatus, and that further elucidation of ribosome biogenesis dynamics in chondrogenic differentiation models will enable an improved understanding of ribosome function during chondrocyte cell commitment and its role in ribosomopathies presenting with a skeletal development aspect. To explore the involvement of ribosome biogenesis during chondrogenic differentiation, we investigated the expression dynamics of factors involved in ribosome biogenesis during *in vitro* ATDC5 chondrogenic differentiation and determined whether protein translation activity adapts to different phases of chondrogenic differentiation.

## 2 Materials and methods

### 2.1 Chondrogenic differentiation of ATDC5 cells

ATDC5 cells [21] (RIKEN BRC, Japan) were cultured in a humidified atmosphere at 37 °C, atmospheric O_2_ concentration (∼20%) and 5% CO_2_ in proliferation medium (Dulbecco's Modified Eagle Medium (DMEM)/F12 (Invitrogen, Carlsbad, CA, USA), 5% fetal calf serum (FCS)(Sigma-Aldrich, St. Louis, MO, USA) and 1% antibiotic/antimycotic (Penicillin-Streptomycin, Invitrogen). Cells were plated at 6400 cells/cm^2^. After 24 h chondrogenic differentiation was initiated with differentiation medium (proliferation medium supplemented with 10 μg/ml insulin (Sigma-Aldrich), 10 μg/ml transferrin (Roche, Basel, Switzerland) and 30 nM sodium selenite (Sigma-Aldrich)). Differentiation medium was refreshed every two days for the first 10 days, and each day after day 10, until cells were harvested at indicated time points.

### 2.2 Real-time quantitative PCR (RT-qPCR)

RNA isolation was undertaken using TRIzol reagent (Invitrogen). RNA was precipitated with isopropanol (30 min, −80 °C) and centrifuged for 30 min at 20,000×*g*, 4 °C. RNA pellets were washed with 80% ethanol and potential DNA contamination was removed by DNase I (Roche) treatment (1 h, 37 °C). After subsequent ethanol precipitation, RNA was dissolved in 15 μL DNase/RNase free water (Eurogentec, Seraing, Belgium). RNA quantity and purity were determined spectrophotometrically (Biodrop, Isogen Life Sciences, Utrecht, the Netherlands). DNA-free total RNA was reverse transcribed using standard procedures and random hexamer priming as described previously [22]. RT-qPCR was performed in 96-well optical plates. For each cDNA sample a mix was prepared consisting of Mesagreen qPCR Mastermix Plus for SYBR Green (Eurogentec) and 300 nM forward and reverse oligonucleotides. Serially diluted standard curves were utilized to quantify gene expression in the samples. A Bio-Rad CFX96 Real-Time PCR Detection System was used for amplification using the following protocol: denaturation at 95 °C for 5 min, followed by 50 cycles of amplification (15 s 95 °C and 45 s 60 °C) followed by a dissociation curve. Data were analyzed using Bio-Rad CFX Manager Software version 3.1, based on the relative quantification of the expression of the target gene normalized to β-Actin housekeeping gene. snoRNA expression was determined by RT-qPCR according to Peffers et al. (2021) [23], and expression was normalized to 5S rRNA expression. Primer sequences are provided in Supplementary Table 1.

### 2.3 Immunoblotting

Cells were washed three times with 0.9% NaCl and subsequently lysed in RIPA buffer (150 mM NaCl, 1% NP-40, 0.5% sodium deoxycholate, 0.1% SDS, 50 mM Tris pH 8.0, 5.0 mM EDTA pH 8.0, 0.5 mM dithiothreitol (DTT) and 1 mM phenylmethylsulfonylfluoride (PMSF)). Samples were homogenized by sonication (Soniprep 150 MSE) on ice using the following protocol: 14 cycles of 1 second sonication followed by a 1 second interval, amplitude 10. Cell debris was removed by means of centrifugation at 15,000 RPM for 10 min at 4 °C. Total protein concentration was determined with a BCA assay (Sigma-Aldrich). Samples were separated by gel electrophoresis and transferred to nitrocellulose membranes by electroblotting. The following primary antibodies were used for immunodetection: Mouse monoclonal anti-Dyskerin (C-11, Santa Cruz, Dallas, TX, USA, #SC-365731; 1:200 dilution), rabbit monoclonal anti-Fibrillarin (EPR10822(B), Abcam, Cambridge, UK, #AB154806; 1:1000 dilution), mouse monoclonal anti-UBF1 (F-9, Santa Cruz #SC-13125; 1:250 dilution), rabbit polyclonal anti-Sox9 (Abcam #AB3697; 1:100 dilution) and mouse monoclonal anti-Puromycin (12D10, Merck Millipore, Billerica, MA, USA, #MABE343; 1:1000 dilution). As a control mouse monoclonal anti-Histone H3 (Abcam #24834; 1:1000 dilution) or mouse monoclonal anti-α-Tubulin (B-5-1-2, Sigma-Aldrich #T6074; 1:10,000 dilution) were used. HRP-conjugated polyclonal rabbit anti-mouse or swine anti-rabbit (Dako) were applied as a secondary antibody and the bound antibodies were detected by enhanced chemiluminescence (ECL) (Bio-Rad Chemidoc XRS+).

### 2.4 SYBR green assay for DNA content quantification

Ribosomal RNA content per cell was quantified by calculating 18S, 5.8S and 28S rRNA RT-qPCR signals in six biological replicates relative to the DNA content. DNA concentrations in equal volumes of papain digestion buffer (100 mM phosphate buffer (NaH_2_PO_4_ (VWR, Amsterdam, the Netherlands) and Na_2_HPO_4_ * 2H_2_O (VWR), pH 6.5), 5 mM l-cysteine*HCl (Sigma-Aldrich), 5 mM EDTA (Ethylenediaminetetraacetic acid)(VWR), 33.33 μg/μl papaine (Sigma-Aldrich)) were determined in samples from day 0, 7 and 14 of ATDC5 chondrogenic differentiation using the SYBR Green assay (Invitrogen). Prior to measurement, samples were diluted in TE (Tris-EDTA) buffer (10 mM Tris/HCl pH 8 and 1 mM EDTA; day 0; 1:100 dilution, day 7 and day 14; 1:1000 dilution). A serially diluted standard curve (0.016–4 μg/ml) of calf thymus genomic control DNA (Invitrogen) in TE buffer was included to quantify the DNA concentration in the samples. Standards were prepared to contain the same amount of papain digestion buffer as the samples. SYBR Green was diluted 10,000 times in TE buffer and 100 μl was added to 100 μl of the above prepared samples and standards. After 10 min incubation fluorescence was determined using a Spectramax M2E (Molecular Devices, Sunnyvale, CA, USA) microplate reader with an excitation of 488 nm and an emission of 522 nm and DNA concentration was calculated using the standard curve.

### 2.5 Determination of active translation by puromycin incorporation

Prior to harvesting at day 0, 3, 7, 10 and 14, ATDC5 cells were incubated with 10 μg/ml puromycin (Sigma-Aldrich) in normal proliferation (day 0) or differentiation medium (day 3, 7, 10 and 14) for 15 min at 37 °C, 5% CO_2_ in a humidified atmosphere. After puromycin was incorporated for exactly 15 min, cells were washed twice with 0.9% NaCl and harvested for immunoblotting with RIPA lysis buffer (see section Immunoblotting). The puromycin signal of the whole lane (volume intensity; vol. INT) was quantified for each sample and normalized for the quantified anti-α-TUBULIN signal (housekeeper) using Bio-Rad Image Lab Software 5.2.1.

### 2.6 snoRNA sequencing

Library preparation, RNA sequencing and bioinformatic analysis was conducted by Exiqon A/S (Vedbaek, Denmark). SnoRNA expression during ATDC5 differentiation was analyzed by RNA sequencing of triplicate samples from day 0 (progenitor stage), day 7 (chondrogenic stage) and day 14 (hypertrophic stage). Total RNA was isolated using a mirVana kit (Thermofisher Scientific) and 5 μg RNA from each sample was supplied to Exiqon A/S for analysis. Total RNA integrity (RIN) was confirmed (all values between 8 and 10). RNA was decapped using Tobacco Acid Pyrophosphatase and 1 μg of decapped total RNA of each sample was converted into RNA sequencing libraries using NEBNext library generation kit (New England Biolabs, Ipswich, MA, USA). Each individual RNA sample had adapters ligated to its 3′ and 5′ ends and was converted to cDNA. Then, the cDNA was pre-amplified with specific primers containing sample specific indexes (Exiqon). After a 15 cycle pre-PCR the libraries were purified on QiaQuick column (Qiagen, Hilden, Germany) and the insert efficiency was evaluated on a Bioanalyzer 2100 instrument (Agilent, Santa Clara, CA, USA) on a high sensitivity DNA chip (Agilent). The cDNA libraries were size fractioned on a LabChip XT (Caliper Life Sciences, Waltham, MA, USA) and 15–200 bp inserts were excised according to the manufacturer's protocol (Caliper). Samples were then quantified using qPCR and a concentration standard. Based on the quality of the inserts and the concentration measurements the libraries were pooled in equimolar concentrations. The library pool was quantified with qPCR and an optimal concentration of the library pool (Illumina) was used to generate the clusters on the surface of a flow cell before sequencing (using v3 sequencing methodology according to the manufacturer's instructions). Libraries were then sequenced on an Illumina MiSeq instrument, yielding 50 nt single-ended reads of high quality (Q-score above 20). An average of 5 million reads was obtained for each sample. After filtering and normalization, using the trimmed mean of the M-values method (read length distribution after filtering of the adapters: 20–200 bp), based on log-fold and absolute gene wise changes in expression levels between samples [24], reads that mapped to snoRNAs were analyzed for differential expression. The reads were aligned to Ensembl GRCm38.p2 mouse genome reference sequences which contains annotated snoRNA features. The counting and annotation was then done on the mapped data and the count values were used as snoRNA expression measurements for the differential expression analysis. The processes and technical details of the analysis included: assessing data variation and detecting outlier samples through comparing variations of within and between sample groups and correlation analysis; formulating data variation using negative binomial distributions and estimation of dispersion using a quantile-adjusted conditional maximum likelihood (qCML) estimator. P-values for significantly differential expressed snoRNAs were estimated by an Fisher's exact test (F-test) on the negative binomial distribution. The Benjamini-Hochberg procedure was applied to control the false discovery rate (FDR) at α = 0.05. Differential regulation was calculated as log(fold change)(LogFC) of expression levels between two groups. Due to limited number of differential expressed snoRNAs using the FDR values, a snoRNA was considered to be differentially expressed if it was significant in the F-test (p < 0.05) and logFC > 1 or < −1. RNA sequencing data have been deposited in the ArrayExpress database at EMBL-EBI (www.ebi.ac.uk/arrayexpress) under accession number E-MTAB-10529.

### 2.7 Polysome fractionation

Polysome fractionation was carried out as described previously [25]. Three 15 cm plates with ATDC5 cells were used to generate a single sample at day 0, two plates at day 7 and one plate at day 14, and this was repeated four times to generate n = 4 biological replicates. At the day of sample collection, cells were pre-treated for 5 min with 100 μg/ml Cycloheximide (Sigma), washed twice in 0.9% NaCl with cycloheximide and collected by scraping with a rubber policeman in cold 0.9% NaCl. Pelleted cells were lysed for 10 min in 1.8 ml polysome extraction buffer (20 mM Tris-HCl (pH7.5), 100 mM KCl, 5 mM MgCl_2_, 0.5% Nonidet P-40, 100 μg/ml Cycloheximide, complete protease inhibitor cocktail (Roche) and RNasin (Promega, 40U/ml)) on ice. Nuclei and cellular debris were removed by centrifugation at 12.000×*g* for 10 min at 4 °C and 9/10th of the total volume was transferred to fresh tubes and measured spectrophotometrically. Ten percent input was set aside and stored at −80 °C. Linear 10–50% sucrose gradients were made using the Gradient Master (BioComp) in SW41 ultracentrifuge tubes (Seton). A fixed amount of 160 μg cytoplasmic extract was loaded to each gradient, for each sample in the same volume. Gradients were run on a Beckman L60 ultra-centrifuge at 39.000 rpm for 1.5 h at 4 °C with max acceleration and deceleration 9. Samples were fractionated into 24 × 0.5 ml fractions using a Piston Gradient fractionator (BioComp) and fraction collector (Gilson FC203B) with continuous A260 monitoring (Triax FC-1).

### 2.8 Statistics in other than snoRNA sequencing

Statistical significance was determined by two-tailed student t-tests using Graphpad PRISM 5.0 (La Jolla, CA, USA). Error bars in graphs represent mean ± standard deviation. Significance for all tests was set at p ≤ 0.05.

## 3 Results

**Fig. 1:**
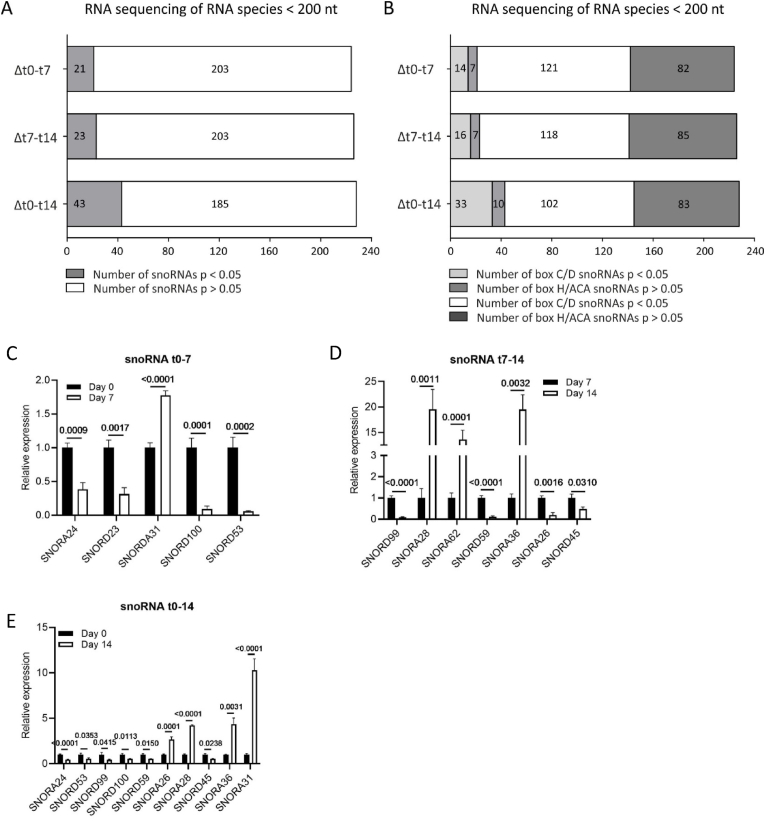
Differential expression of snoRNAs during different phases of chondrogenic differentiation. ATDC5 cells were differentiated in the chondrogenic lineage and RNA sequencing of RNA species <200 nt was performed at samples from day 0, 7 or 14 days in differentiation. A) The total number of snoRNAs and differentially expressed (p-value <0.05, logFC > 1 or < −1) snoRNAs identified with RNA sequencing between day 0 and day 7 (initiation of chondrogenic differentiation), day 7 and day 14 (hypertrophic differentiation) and day 0 and day 14 (progenitor versus hypertrophic chondrocyte). B) Subdivision between box C/D versus box H/ACA snoRNAs in differentially and non-differentially expressed snoRNAs as identified by RNA sequencing. C-E) In samples from day 0, 7 and 14 in ATDC5 differentiation snoRNA expression was determined by RT-qPCR according to Peffers et al. (2020) [23]. Gene-expression was normalized to 5S rRNA expression. Data (mean + standard deviation; n = 6 biological replicates) is depicted as fold change relative to t = 0 (C and E) or day 7 (D). For statistical evaluation an independent samples *t*-test was performed between each time point. p-values are indicated.

Chondrogenic differentiation of ATDC5 was performed and differentiation was confirmed on samples from day 0, 4, 7, 10 and 14 in differentiation by measuring the expression of chondrogenic marker genes (Supplementary Fig. 1). To identify snoRNAs that are regulated during chondrogenic differentiation, we performed RNA sequencing (<200 nt) of samples from these ATDC5 cultures at day 0, 7 and 14. Expression of at least 228 different snoRNA species was detected (Fig. 1 and Supplementary Table 2). snoRNAs are classified as C/D box small nucleolar RNAs (SNORDs) or H/ACA box snoRNAs (SNORAs), based on conserved sequence elements [26]. We found 21 different snoRNAs differentially expressed between day 0 and day 7 in differentiation (Fig. 1A; Δt0-t7) of which 14 were box C/D snoRNAs and 7 were box H/ACA snoRNAs (Fig. 1B; Δt0-t7). Differential expression of 23 snoRNAs was detected between day 7 and 14 (Fig. 1A; Δt7-t14) of which 16 were box C/D snoRNAs and 7 box H/ACA snoRNAs (Fig. 1B; Δt7-t14). In addition, differential expression of 43 snoRNAs was found when comparing day 0 with day 14 data (Fig. 1A; Δt0-t14), with 33 of which were box C/D snoRNAs and 10 were box H/ACA snoRNAs (Fig. 1B; Δt0-t14). The top 10 of the differentially expressed snoRNAs and their putative ribosomal RNA (rRNA) targets (2′*O*-ribose methylation or pseudouridylation of specific rRNA nucleotides) is presented in Table 1 (for the full overview see Supplementary Table 3. Validation of differentially expressed snoRNAs by RT-qPCR is presented in Fig. 1C–E. The majority of snoRNAs are involved in the post-transcriptional modification of rRNAs [26]. To map which rRNA domains are putative targets of the differentially expressed snoRNAs identified in our small RNA sequencing analysis, we plotted the rRNA targets of the differentially expressed snoRNAs on the 2D rRNA structure of 18S (Fig. 2A), 5.8S and 28S rRNAs (Fig. 2B). This revealed that differentially expressed snoRNAs, amongst others, target post-transcriptional modifications in the vicinity of the 18S rRNA decoding center (DC; Fig. 2A, open black circles indicate the five helices of the 18S DC) and of the 28S rRNA peptidyl transferase center (PTC; Fig. 2B, open black circles indicate the helices of the 28S PTC).

**Table 1:** Top 10 significantly differentially expressed snoRNAs between different phases of ATDC5 chondrogenic differentiation.

Δ day 0–7	Box	RNA target	LogFC	p-value
SNORA24	H/ACA	18S rRNA U863 +U609	−2.84	0.001
SNORD1C	C/D	28S rRNA G4362	−2.27	0.001
SNORD101	C/D	Unknown/orphan	−1.51	0.006
SNORD1A	C/D	28S rRNA G4362	−2.20	0.007
SNORD1B	C/D	28S rRNA G4362	−1.68	0.007
SNORD80	C/D	28S rRNA A1521 G1612	−2.77	0.010
SNORA66	H/ACA	18S rRNA U119	−1.53	0.013
SNORA12	H/ACA	U6 snRNA U40	−1.53	0.013
SNORD23	C/D	Unknown/orphan	−2.27	0.013
SNORA31	H/ACA	18S rRNA U218 28S rRNA U3713	1.51	0.014
Δ day 7–14	Box	RNA target	LogFC	p-value
SNORD103	C/D	18S rRNA G601	−2.14	0.001
SNORD30	C/D	28S rRNA A3804	−2.03	0.001
SNORD99	C/D	28S rRNA A2774	−2.00	0.001
SNORD36	C/D	18S rRNA A668 28S rRNA A3703	−1.98	0.001
SNORD55	C/D	28S rRNA C2791	−1.92	0.001
SNORD52	C/D	28S rRNA U3904	−1.81	0.002
SNORD66	C/D	18S rRNA C1272	−1.88	0.006
SNORD21	C/D	28S rRNA G1303	−1.56	0.006
SNORA28	H/ACA	18S rRNA U815 U866	1.48	0.009
SNORA40	H/ACA	18S rRNA U1174 28S rRNA U4546	1.72	0.010
Δ day 0–14	Box	RNA target	LogFC	p-value
SNORD36	C/D	18S rRNA A668 28S rRNA A3703	−3.18	4.79E-07
SNORA24	H/ACA	18S rRNA U609 U863	−3.50	2.85E-06
SNORD101	C/D	Unknown/orphan	−2.36	3.39E-05
SNORD1C	C/D	28S rRNA G4362	−3.25	5.93E-05
SNORD55	C/D	28S rRNA C2791	−2.08	2.69E-04
SNORA30	H/ACA	28S rRNA U4643	2.45	2.99E-04
SNORD1B	C/D	28S rRNA G4362	−2.49	0.001
SNORD53	C/D	28S rRNA C3848	−1.87	0.001
SNORD42A	C/D	18S rRNA U116	−1.80	0.002
SNORD2	C/D	28S rRNA G1509	−1.86	0.002

The top 10 ATDC5 differentiation phase-dependent significantly differentially expressed snoRNAs are indicated per contrast (Δt0-t7, Δt7-t14, Δt0-t14). rRNA target information was acquired from snoRNABase [8]. The complete list of significantly differentially expressed snoRNAs is presented in Supplementary Table 3.

**Fig. 2A:**
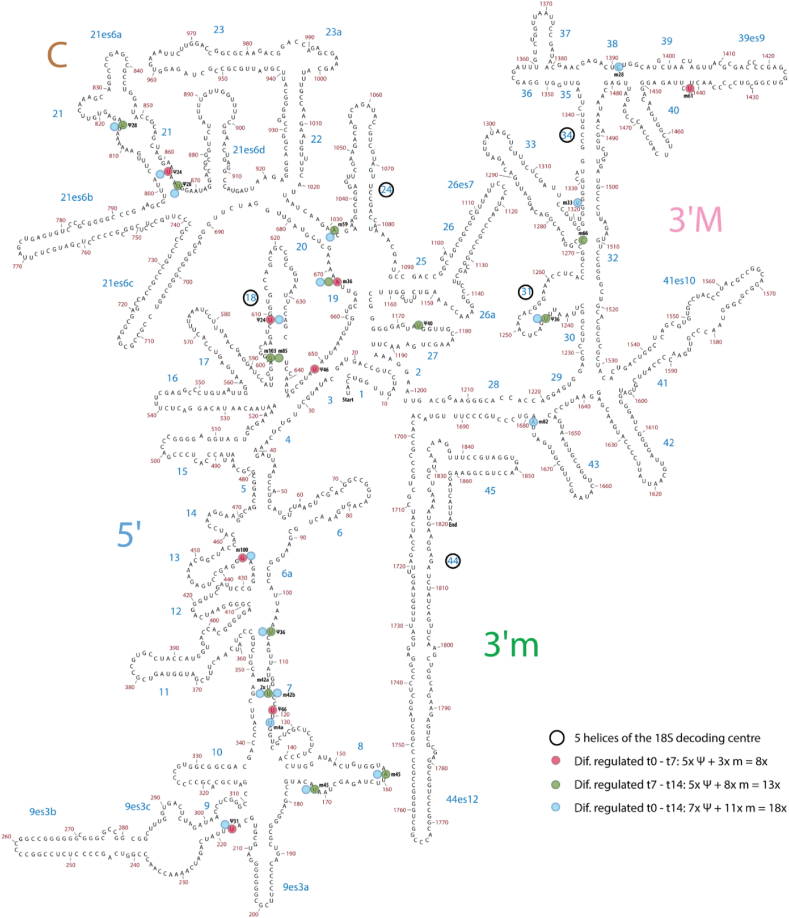
Target sites of differentially expressed snoRNAs in the 18S small ribosomal subunit. Target sites of differentially expressed snoRNAs between day 0 and 7 are visualized in pink; Δ day 7–14 in green and Δ day 0–14 in blue. Specific snoRNAs are indicated (m = 2′-O ribose methylation, Ψ = pseudouridylation). Open black circles indicate the 5 helices of the 18S decoding centre. SNORA40 in helix 27 is indicated by the black box. Human secondary rRNA structure was adapted from Apollo Chemistry Gatech Ribovision Ribosome Visualization Suite [9].

**Fig. 2B:**
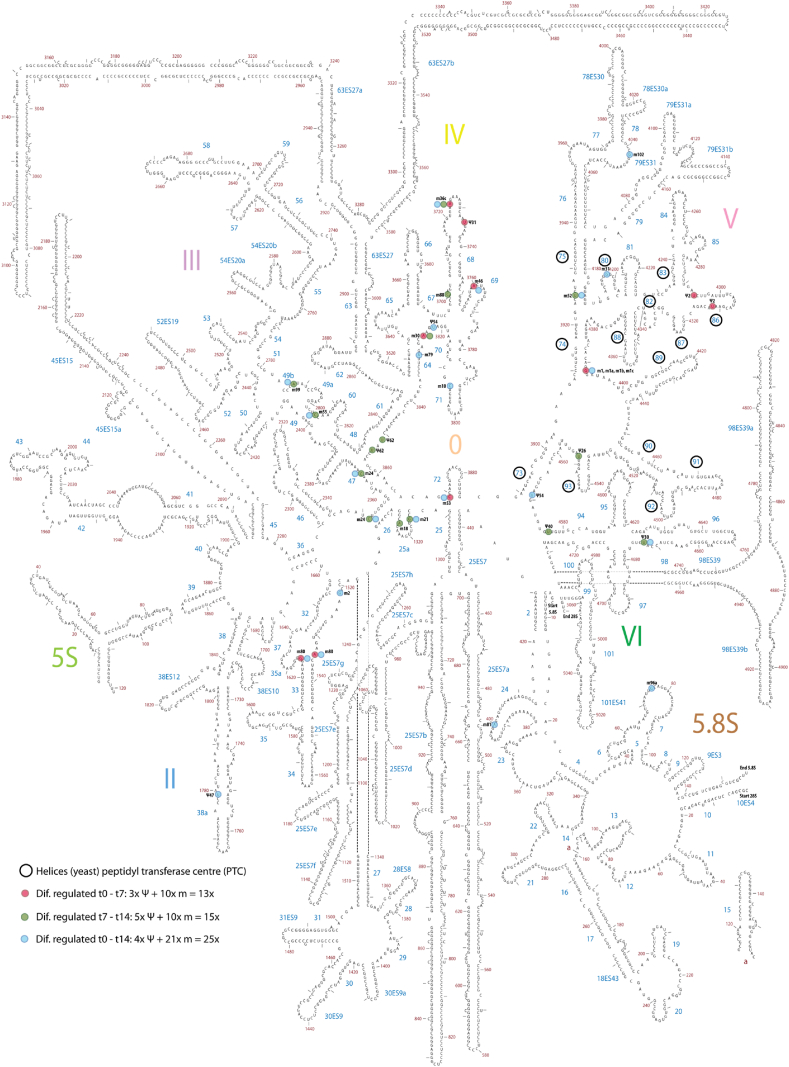
Target sites of differentially expressed snoRNAs in the large ribosomal subunit (5S, 5.8S and 28S). Target sites of differentially expressed snoRNAs between day 0 and 7 are visualized in pink; Δ day 7–14 in green and Δ day 0–14 in blue. Specific snoRNAs are indicated (m = 2′-O ribose methylation, Ψ = pseudouridylation). Open black circles indicate the helices of the 28S peptidyl transferase center. Human secondary rRNA structure was adapted from Apollo Chemistry Gatech Ribovision Ribosome Visualization Suite [9].

**Fig. 3:**
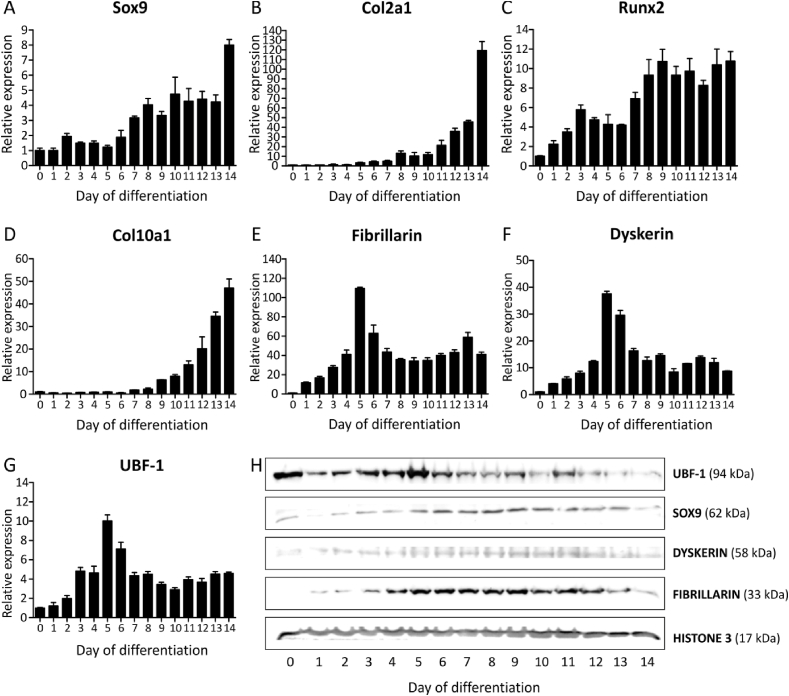
Fibrillarin, Dyskerin and UBF-1 expression adapts to the differentiation status of ATDC5 cells. ATDC5 cells were differentiated in the chondrogenic lineage for 14 days. A-D) Different stages of chondrogenic differentiation were confirmed by measuring gene expression of *Sox9*, *Col2a1*, *Runx2* and *Col10a1*. H/SOX9) SOX9 protein expression was also determined. E-G) Gene expression of Fibrillarin, Dyskerin and UBF-1 was determined. Data (A–G) is depicted as fold induction relative to t = 0. Data was normalized to *β-actin* and represents the average value of four biological replicates plus standard deviation. H) Protein expression of FIBRILLARIN, DYSKERIN and UBF-1 was detected on the same blot. HISTONE 3 was used as a housekeeper.

SnoRNAs site-directionally guide the modification of their rRNA targets by sequence complementarity. However, the 2′*O*-ribose methylase and pseudouridylase activity that ultimately leads to the rRNA post-transcriptional modifications is carried out by fibrillarin and dyskerin, respectively. With fibrillarin being a core component of Box C/D snoRNPs (Small nucleolar ribonucleoproteins) and dyskerin of Box H/ACA snoRNPs. We therefore asked whether the expression of fibrillarin and dyskerin also alters as a function of chondrogenic differentiation. We performed a high-resolution sampling of ATDC5 chondrogenic differentiation. Conforming chondrogenic differentiation, the mRNA expression of Sox9 (SRY-Box Transcription Factor 9), Col2a1 (Collagen Type II Alpha 1 Chain), Runx2 (Runt-related transcription factor 2) and Col10a1 (Collagen Type II Alpha 1 Chain) (Fig. 3A–D) was induced over a course of 14 days. This was further confirmed by Sox9 immunoblotting (Fig. 3H). Gene expression of fibrillarin and dyskerin was measured in these samples. We detected a steady increase over-time of the mRNA expression of both fibrillarin and dyskerin, with a marked peak-expression at day 5 and 6 in differentiation, followed by maintenance of elevated fibrillarin and dyskerin expression as compared to non-differentiated (t = 0) ATDC5 (Fig. 3E/F). The steady increase of fibrillarin and dyskerin expression was also detected at the protein level, albeit more evident for fibrillarin (Fig. 3H). Together, these data indicate that during the course of chondrogenic differentiation snoRNAs are differentially regulated and the expression of their core proteins fibrillarin and dyskerin depends on the phase of chondrogenic differentiation.

**Fig. 4:**
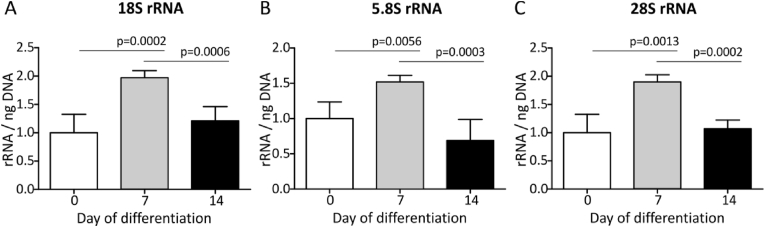
18S, 5.8S and 28S rRNA content per cell adapts to the differentiation status of ATDC5 cells. ATDC5 cells were differentiated in the chondrogenic lineage for 0, 7 or 14 days. A-C) 18S, 5.8S and 28S rRNA content per cell was quantified by calculating 18S, 5.8S and 28S rRNA RT-qPCR signals in six biological replicates relative to the DNA content in six biological replicates as quantified by SYBR green assay. Data (mean + standard deviation) is depicted as fold change relative to t = 0. For statistical evaluation an independent samples *t*-test was performed between each consecutive time point. p-values are indicated.

**Fig. 5:**
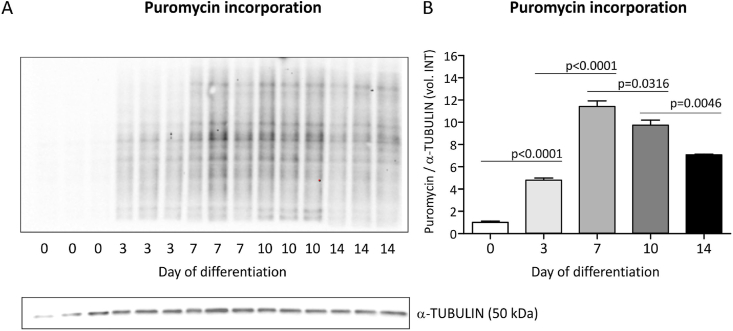
Active translation differs according to the differentiation status of ATDC5 cells. To determine active protein synthesis, ATDC5 cells were labeled with puromycin 15 min prior to harvesting. Proteins were separated by gel electrophoresis and puromycin was detected. The experiment was performed with six biological replicates per time point. Here, three representative biological replicates per time point are shown (i.e. one of two Western blots). A) Whole lane puromycin signal (volume intensity; vol. INT) was quantified in all six biological replicates per time point and corrected for the quantified anti-α-TUBULIN signal (housekeeper) (B) using Bio-Rad Image Lab Software 5.2.1. Data (mean + standard deviation) is depicted as fold change relative to t = 0. For statistical evaluation an independent samples *t*-test was performed between each consecutive time point. p-values are indicated.

**Fig. 6:**
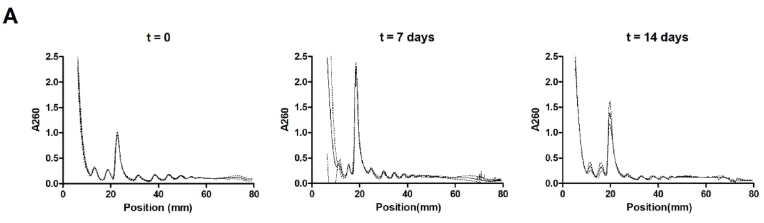
Polysome fractionation of differentiating ATDC5 cells. Cytoplasmic extracts were generated at day 0, 7 and 14 of ATDC5 differentiation and equal amounts were ran on freshly prepared 10–50% sucrose gradients (n = 4/time point). After ultracentrifugation, sucrose gradients were fractionated with continuous recording of A260. Position (mm) indicates the position (depth) in the centrifugation tube with “0” representing the top and “80” the bottom of the centrifugation tube.

Taking the changing snoRNA expression landscape into consideration and the fact that cartilaginous (collagenous) extra-cellular matrix synthesis is induced during ATDC5 chondrogenic differentiation [16,27], we next questioned whether cellular protein translation capacity is regulated during different phases in ATDC5 chondrogenic differentiation. We measured the expression of 18S rRNA, 5.8S rRNA and 28S rRNA at 0, 7 and 14 days in ATDC5 differentiation. Data show that the expression of these rRNAs changes over-time, with significantly elevated levels specifically at day 7, but not at day 14 in differentiation (Fig. 4), compared to undifferentiated (t = 0) ATDC5. In concert with these elevated rRNA levels at day 7 in ATDC5 differentiation, we found that the mRNA and protein expression of key rRNA transcription factor UBF-1 [28] peaked at day 5/6 (Fig. 3G/H). Total protein translation capacity increased over-time during the course of ATDC5 chondrogenic differentiation, reaching its peak activity at day 7 in differentiation (Fig. 5). At later time points, translation capacity remained induced, albeit at lower activity than at day 7. The protein translation activity of differentiating ATDC5 was further investigated by polysome profiling. Sucrose gradient polysome profiling of cytoplasmic extracts of undifferentiated (day 0), and day 7 or day 14 differentiated ATDC5 revealed a strong increase in the monosomal ribosome fraction (∼20 mm) at day 7 in differentiation when compared to undifferentiated ATDC5 cells, while the polysomal distribution (>20 mm) was largely unaltered (Fig. 6). When progressing further into differentiation to day 14, the increased monosomal fraction observed at day 7 was reduced to a level that was still greater when compared to day 0 (Fig. 6). The polysomal fraction was again not altered, although an overall reduction in peak height can be observed between day 7 and 14 in differentiation. The density gradient dynamics observed in the monosomal fractions are in concert with the dynamics observed in rRNA expression total protein translation capacity over the course of differentiation (Fig. 5).

## 4 Discussion

Cell differentiation requires major changes in the cellular proteome to accommodate the cell specialization process [29]. Specifically, in the case of chondrogenic differentiation we expected that the *de novo* formation of the cartilaginous extracellular matrix demands a major proteomic effort from the cell. In keeping with this notion, rRNA levels were found to depend on the stage of chondrogenic differentiation. Specifically at day 7 in ATDC5 chondrogenic differentiation the rRNA expression levels were significantly higher, while rRNA levels were decreased again at day 14. This corresponded with increased protein translation and highest monosomal peaks in polysome profiling at day 7. These timings represent separate chondrogenic differentiation stages, with day 7 being early, highly proliferative [30], and predominantly associated with extracellular matrix production, rich in type II collagen and aggrecan [16,27]. On the other hand, day 14 represents end-stage differentiation with expression of terminal differentiation markers type X collagen and Alkaline phosphatase. One of the central transcription factors of RNA polymerase I-driven (RNAPI) 47S rDNA transcription is UBF-1 (Upstream Binding Transcription Factor) [28]. And, although RNAPI activity is regulated via UBF-1 at the post-translational level (phosphorylation of UBF-1), the here observed dynamics in UBF-1 expression during the course of chondrogenic differentiation strongly indicate that the increased rRNA levels at day 7 are, at least in part, the result of an increased transcription of the 47S rDNA gene. In concert with the peak rRNA expression observed at day 7 in ATDC5 differentiation, total cellular translation capacity also reached its highest level at day 7 in differentiation, emphasizing that rRNA levels and translational capacity are functionally connected in the chondrogenic differentiation program. Chondrogenic differentiation *in vivo* and *in vitro* [31] largely depends on IGF-1 (Insulin-like growth factor 1) signaling and indeed *in vitro* ATDC5 chondrogenic differentiation is also stimulated by insulin [21]. Insulin is one of the best-studied drivers of 47S rDNA transcription and acts via UBF-1-dependent activation of the RNAPI machinery [32]. In addition, mTOR (mechanistic target of rapamycin) and 4E-BP (4E-binding protein) activity are also induced by insulin and determine ribosome translational activity [33]. Together we therefore expect that during chondrogenic differentiation, rRNA levels and protein translational activity are regulated by insulin signaling to meet the increased demand for cartilaginous extracellular matrix production enabling the developing chondrocyte to translationally adapt to the cell specialization process.

Translational activity of the ribosome depends on many factors, with post-transcriptional modification of the rRNAs being pivotal in the basic biogenesis of the ribosome, supporting rRNA structural stability, ribosomal protein association and maturation of crucial ribosome functional regions, like the peptidyl transferase center (PTC) and the decoding center [14,34]. While hundreds of different rRNA post-transcriptional modifications are being guided by a great number of site-specific snoRNAs [35], the core enzymatic activities responsible for these post-transcriptional modifications are fibrillarin [36] (for 2′*O*-ribose methylation) and dyskerin (for pseudouridylation) [37]. Almost simultaneously with elevated rRNA levels at day 7 in ATDC5 chondrogenic differentiation, the expression of fibrillarin and dyskerin synchronously reached their highest levels from day 5–6 and onward. This observation may be explained by an increased *de novo* synthesized rRNA pool that requires post-transcriptional modification. In addition, alterations in fibrillarin or dyskerin expression have been shown to provoke changes in ribosome translational characteristics. Knockdown of fibrillarin caused a reduction in global protein translation, with a specific reduction in IRES-dependent protein translation [36], and p53-dependent expression of fibrillarin has been shown to regulate IRES-dependent translation and translational fidelity [12]. Reduction of dyskerin levels also altered IRES-dependent translation, but with IRES-specific effects [11,38]. It is unknown at this point whether these alterations in fibrillarin and dyskerin levels change translational characteristics via snoRNA-specific actions. Apart from the apparent global increased need for rRNA post-transcriptional modification capacity, we established that the specific snoRNA expression landscape during ATDC5 chondrogenic differentiation changes, depending of the differentiation stage. This indicates that apart from the total post-transcriptional maturation of a larger cellular rRNA pool, also the position of various specific rRNA post-transcriptional modifications may be adapting to the chondrogenic differentiation stage. We cannot rule out, however, that measuring the rRNAs and snoRNAs at a higher time point resolution during chondrogenic differentiation of ATDC5 cells might show a different expression dynamic that will provide more insight into this matter. There is limited literature reporting on the differential expression of snoRNAs in models for cell differentiation. In neural differentiation from embryonic stem cells, specific snoRNA species were found to be differentially expressed, depending on the differentiation stage [10]. In hematopoietic development, many snoRNA was differentially expressed in a lineage specific pattern [39]. SnoRNA species were also expressed in a differentiation stage-dependent manner during hepatic differentiation of induced pluripotent stem cells [40]. Apart from a number of non-canonical snoRNAs (like SNORD101, SNORD23, SNORA73 and others) that are not involved in rRNA post-transcriptional modification, the differentiation stage-depend dynamics in canonical snoRNA expression predicts a significant degree of rRNA post-transcriptional modification regulation during cell differentiation. A number of snoRNAs with differentiation stage-dependent expression dynamics in snoRNA expression during ATDC5 chondrogenic differentiation are guiding post-transcriptional modification of rRNA sites in the critical ribosome regions like the decoding center, the peptidyl transferase center and E-site. For example, SNORA40 (modifying helix 27 in the 18S rRNA decoding center in yeast [41]), SNORD46 (modifying helix 69 in 28S rRNA [42,43]) and SNORD36C and SNORA31 (modifying helix 68 in the 28S rRNA ribosome's E-site [44]). A recent seminal work showed regulation of rRNA 2′*O*-ribose methylation during mouse development and accompanying guide snoRNAs and highlights the relevance of ribosome heterogeneity during cell development [45]. In our ATDC5 chondrogenic differentiation model it remains to be determined whether the rRNA target sites of the differentially expressed snoRNAs are actually post-transcriptionally modified in a differentiation stage-specific manner and how this may influence differentiation stage-specific rRNA PTM-based ribosome heterogeneity.

In conclusion, our data show that chondrogenic differentiation is associated with significant regulation of mechanisms involving ribosome biogenesis and translation activity. Differentiation-phase specific expression of snoRNAs suggests that specific snoRNAs may modulate the chondrocyte's developing phenotype via an rRNA PTM-based ribosome heterogeneity mechanism, thereby potentially facilitating the observed dynamics in translational activity impacting the course of chondrogenic differentiation. Future work is expected to uncover the extent of ribosome heterogeneity and regulation in cellular differentiation and its potential implications for human disease.

## Contribution to the field statement

Ribosomes are universally responsible for translating mRNAs into protein. Ribosome synthesis is integrated with many cellular processes including proliferation, differentiation and oncogenic events. Chondrogenic progenitor cell proliferation and differentiation require a high cellular translational capacity to facilitate cartilaginous extracellular matrix production. However, how ribosome biogenesis is integrated in chondrogenic differentiation remains to be determined. Further elucidation of ribosome biogenesis dynamics in developmental models, will enable improved understanding of ribosome function changes during cell commitment and their role in human disease.

## Data availability statement

RNA sequencing data have been deposited in the ArrayExpress database at EMBL-EBI (www.ebi.ac.uk/arrayexpress) under accession number E-MTAB-10529.

## CRediT authorship contribution statement

**Mandy M.F. Steinbusch:** Methodology, Funding acquisition, Formal analysis, Interpretation of data, Writing – original draft, revising paper critically, approval of the submitted and final versions. **Guus G.H. van den Akker:** Methodology, Funding acquisition, Formal analysis, Interpretation of data, Writing – original draft, revising paper critically, approval of the submitted and final versions. **Andy Cremers:** Funding acquisition, Formal analysis, Interpretation of data, revising paper critically, approval of the submitted and final versions. **Adhiambo M.A. Witlox:** Methodology, Interpretation of data, revising paper critically, approval of the submitted and final versions. **Heleen M. Staal:** Methodology, Interpretation of data, revising paper critically, approval of the submitted and final versions. **Mandy J. Peffers:** Funding acquisition, Formal analysis, Interpretation of data. **Lodewijk W. van Rhijn:** Methodology, Interpretation of data. **Marjolein M.J. Caron:** Methodology, Funding acquisition, Formal analysis, Interpretation of data, Writing – original draft, revising paper critically, approval of the submitted and final versions. **Tim J.M. Welting:** Methodology, Funding acquisition, Formal analysis, Interpretation of data, Writing – original draft, revising paper critically, approval of the submitted and final versions, All authors have read and approved the final submitted manuscript.

## Declaration of competing interest

None.
