# Supplementary material for: Adaptation of the protein translational apparatus during ATDC5 chondrogenic differentiation
Source: Noncoding RNA Res. 2022 Feb 20;7(2):55–65. doi: 10.1016/j.ncrna.2022.02.003 (PMC8881200; doi:10.1016/j.ncrna.2022.02.003)
Supplement: Multimedia component 1 [file mmc1.pdf]

Supplementary Material

Adaptation of the protein translational apparatus during ATDC5 chondrogenic differentiation

Mandy M.F. Steinbusch[1], Guus G.H. van den Akker[1], Andy Cremers[1], Adhiambo M.A. Witlox[2], Heleen M. Staal[2], Mandy J. Peffers[3], Lodewijk W. van Rhijn[2], Marjolein M.J. Caron[1†*], Tim J.M. Welting[1,2†]

[1]Laboratory for Experimental Orthopedics, Department of Orthopedic Surgery, Maastricht University, P.O. Box 5800, 6202 AZ, Maastricht, the Netherlands.

[2]Laboratory for Experimental Orthopedics, Department of Orthopedic Surgery, Maastricht University Medical Center, P.O. Box 5800, 6202 AZ, Maastricht, the Netherlands.

[3]Department of Musculoskeletal Biology, Institute of Life Course and Medical Sciences, University of Liverpool, William Henry Duncan Building, 6 West Derby Street, L7 8TX, Liverpool, United Kingdom

[†] These authors share senior authorship

Supplementary Table 1. RT-qPCR primer sequences

**Gene Symbol**	**Forward**	**Reverse**
Actb	CCGAGCGCGAGATCGT	TGGCCATCTCGTTCTCGAA
Col2a1	TGGGTGTTCTATTTATTTATTGTCTTCCT	GCGTTGGACTCACACCAGTTAGT
Col10a1	CATGCCTGATGGCTTCATAAA	AAGCAGACACGGGCATACCT
Dkc1	CCACTCGTTTGGTGAAATCACA	AGCCGGACAATTCCAACATACT
Fbl	TGGAGCCGCATCGTCAT	CAAGGGCATCCTCCTTTCC
Runx2	GACGAGGCAAGAGTTTCACC	GGACCGTCCACTGTCACTTT
5.8S rRNA	CACTCGGCTCGTGCGTCGAT	CGCTCAGACAGGCGTAGCCC
18S rRNA	AGTCCCTGCCCTTTGTACACA	GATCCGAGGGCCTCACTAAAC
28S rRNA	GCCATGGTAATCCTGCTCAGTAC	GCTCCTCAGCCAAGCACATAC
Sox9	AGTACCCGCACCTGCACAAC	TACTTGTAGTCCGGGTGGTCTTTC
Ubf-1	CAGGACCGTGCAGCATATAAAG	GCCTCGCAGCTTGGTCAT
**snoRNA**	**Forward**	**Reverse**
SNORA24	TTGGTACCAGATTCTGACTTGAGC	AATTCTAGAGCTCGAGGCAGG
SNORD23	CAGTTGGGCCATTGCCTGGGG	CGAATTCTAGAGCTCGAGGCAGG
SNORA31	CTTTGTGGCAGTTCAGATTGAATTAG	AATTCTAGAGCTCGAGGCAGG
SNORD100	CTCGCTGAGGAAACTGCATGTCAC	CGAATTCTAGAGCTCGAGGCAGG
SNORD53	GAGATGACACCTTTCTCTTGGC	AATTCTAGAGCTCGAGGCAGG
SNORD99	GACATCTATGGATGAGAAATGCGG	AATTCTAGAGCTCGAGGCAGG
SNORA28	CATGAGACAAGCCGTTATATAGGC	AATTCTAGAGCTCGAGGCAGG
SNORA62	AGTGAGTGGGGTTTGATAGTAACC	AATTCTAGAGCTCGAGGCAGG
SNORD59	AGTTTGCTGAAGCCACACTCAG	AATTCTAGAGCTCGAGGCAGG
SNORA36	GTAATTACTCTATTCATATACTTCG	TAGTTAAGCTTGGTACCGAG
SNORA26	AAACTACACCTCCTCTTTGGATCC	AATTCTAGAGCTCGAGGCAGG
SNORD45	AACCTGATGCAAGTTACAAATTAC	TAGTTAAGCTTGGTACCGAG
5S rRNA	CCTGGGAATACCGGGTGCTGTAG	CGAATTCTAGAGCTCGAGGCAGG

The 5’ to 3’ forward and reverse oligonucleotide sequences (Mus musculus) used for RT-qPCR are listed in the table

Supplementary Table 2. RNA sequencing in ATDC5 differentiation

- ➔ **Excelfile**

Supplementary Table 3. Differentially expressed snoRNAs during different phases of ATDC5 chondrogenic differentiation

**Δ day 0 – 7 snoRNA**	**Box**	**RNA target**	**Count day 0**	**Count day 7**	**LogFC**	**p-value**
SNORA24	H/ACA	18S rRNA	45	7	-2.84	0.001
SNORD1C	C/D	28S rRNA	29	7	-2.27	0.001
SNORD101	C/D	Unknown /	1365	584	-1.51	0.006
SNORD1A	C/D	28S rRNA	14	3	-2.20	0.007
SNORD1B	C/D	28S rRNA	34	12	-1.68	0.007
SNORD80	C/D	28S rRNA	6	1	-2.77	0.010
SNORA66	H/ACA	18S rRNA	32	13	-1.53	0.013
SNORA12	H/ACA	U6 snRNA	185	73	-1.53	0.013
SNORD23	C/D	Unknown /	25	6	-2.27	0.013
SNORA31	H/ACA	18S rRNA	40	128	1.51	0.014
SNORA2	H/ACA	28S rRNA	12	4	-1.71	0.023
SNORD46	C/D	28S rRNA	16913	8541	-1.45	0.024
SNORD100	C/D	18S rRNA	47	23	-1.30	0.026
SNORD61	C/D	18S rRNA	1724	4729	1.18	0.031
SNORD1	C/D	28S rRNA	1870	875	-1.70	0.034
SNORD36	C/D	18S rRNA	1033	593	-1.17	0.036
SNORA73	H/ACA	Unknown /	54	24	-1.53	0.039
SNORD53	C/D	28S rRNA	1952	1124	-1.11	0.040
SNORD50B	C/D	Unknown /	4440	14264	1.36	0.042
SNORD30	C/D	28S rRNA	22986	58998	1.17	0.045
SNORA46	H/ACA	18S rRNA	190	101	-1.23	0.049
**Δ day 7 – 14 snoRNA**	**Box**	**RNA target**	**Count day 7**	**Count day 14**	**LogFC**	**p-value**
SNORD103	C/D	18S rRNA	355	73	-2.14	0.001
SNORD30	C/D	28S rRNA	58998	13859	-2.03	0.001
SNORD99	C/D	28S rRNA	155	34	-2.00	0.001
SNORD36	C/D	18S rRNA	593	144	-1.98	0.001
SNORD55	C/D	28S rRNA	554	132	-1.92	0.001
SNORD52	C/D	28S rRNA	20290	5365	-1.81	0.002
SNORD66	C/D	18S rRNA	7071	1833	-1.88	0.006
SNORD21	C/D	28S rRNA	14514	4580	-1.56	0.006
SNORA28	H/ACA	18S rRNA	151	401	1.48	0.009
SNORA40	H/ACA	18S rRNA	14	43	1.72	0.010
SNORD88	C/D	28S rRNA	11	2	-2.26	0.011
SNORD42B	C/D	18S rRNA	117	34	-1.60	0.012
SNORD24	C/D	28S rRNA	27793	7902	-1.67	0.014
SNORA62	H/ACA	28S rRNA	13	38	1.61	0.020
SNORD3	C/D	Unknown /	1218	220	-2.20	0.020
SNORD59	C/D	18S rRNA	191	69	-1.33	0.028
SCARNA3B	H/ACA	U6 snRNA	2	8	2.07	0.030

SNORA30	H/ACA	28S rRNA	18	49	1.43	0.033
SNORD85	C/D	18S rRNA	44	16	-1.32	0.038
SNORD18	C/D	28S rRNA	4634	1853	-1.27	0.039
SNORA36	H/ACA	18S rRNA	44	99	1.26	0.044
SNORA26	H/ACA	28S rRNA	6	1	-1.87	0.044
SNORD45	C/D	18S rRNA	89488	39837	-1.09	0.049
**Δ day 0 – 14 snoRNA**	**Box**	**RNA target**	**Count day 0**	**Count day 14**	**LogFC**	**p-value**
SNORD36	C/D	18S rRNA	1033	144	-3.18	4.79E-07
SNORA24	H/ACA	18S rRNA	45	5	-3.50	2.85E-06
SNORD101	C/D	Unknown /	1365	312	-2.36	3.39E-05
SNORD1C	C/D	28S rRNA	29	3	-3.25	5.93E-05
SNORD55	C/D	28S rRNA	472	132	-2.08	2.69E-04
SNORA30	H/ACA	28S rRNA	7	49	2.45	2.99E-04
SNORD1B	C/D	28S rRNA	34	7	-2.49	0.001
SNORD53	C/D	28S rRNA	1952	643	-1.87	0.001
SNORD42A	C/D	18S rRNA	834	274	-1.80	0.002
SNORD2	C/D	28S rRNA	22228	6971	-1.86	0.002
SNORD42B	C/D	18S rRNA	134	34	-2.09	0.002
SNORD81	C/D	28S rRNA	14	2	-2.54	0.002
SNORD82	C/D	18S rRNA	41432	15120	-1.76	0.003
SNORD99	C/D	28S rRNA	105	34	-1.80	0.003
SNORD100	C/D	18S rRNA	47	15	-1.83	0.003
SNORA47	H/ACA	28S rRNA	7	35	1.98	0.004
SNORD31	C/D	28S rRNA	758	291	-1.58	0.005
SNORD3B2	C/D	Unknown /	1324	344	-2.03	0.005
SNORD46	C/D	28S rRNA	16913	6417	-1.80	0.005
SNORD96A	C/D	5.8S rRNA	293	109	-1.61	0.005
SNORD102	C/D	28S rRNA	12662	4647	-1.67	0.005
SNORD21	C/D	28S rRNA	10972	4580	-1.61	0.006
SNORD1A	C/D	28S rRNA	14	2	-2.47	0.006
SNORD59	C/D	18S rRNA	191	69	-1.63	0.007
SNORA57	H/ACA	U5 snRNA	22	76	1.59	0.009
SNORD1	C/D	28S rRNA	1870	629	-2.11	0.014
SNORA54	H/ACA	28S rRNA	10	38	1.75	0.016
SNORD10	C/D	U6 snRNA	1612	5003	1.36	0.017
SNORD79	C/D	28S rRNA	54	25	-1.43	0.020
SNORA26	H/ACA	U2 snRNA	14	48	1.52	0.021
SNORD4A	C/D	18S rRNA	67	19	-1.83	0.021
SNORD28	C/D	18S rRNA	6570	2962	-1.35	0.021
SNORA28	H/ACA	18S rRNA	131	401	1.36	0.023
SNORD45	C/D	18S rRNA	76766	39837	-1.20	0.028
SNORD33	C/D	18S rRNA	4316	2211	-1.22	0.029
ScaRNA14	H/ACA	U2 snRNA	2	11	1.87	0.031
SNORD24	C/D	28S rRNA	8268	4279	-1.20	0.032

SNORA36	H/ACA	18S rRNA	34	99	1.32	0.035
SNORD52	C/D	28S rRNA	10449	5365	-1.17	0.041
SNORD86	C/D	Unknown /	217	104	-1.23	0.044
SNORD94	C/D	U6 snRNA	220	569	1.15	0.046
SNORD80	C/D	28S rRNA	6	2	-1.90	0.048
SNORA31	H/ACA	18S rRNA	40	109	1.30	0.049

The ATDC5 differentiation phase dependent significantly differentially expressed snoRNAs are indicated per contrast (Δt0-t7, Δt7-t14, Δt0-t14). For each snoRNA the RNA target is indicated, as well as the counts, fold-differences and significance. RNA target information was acquired from snoRNABase [1].

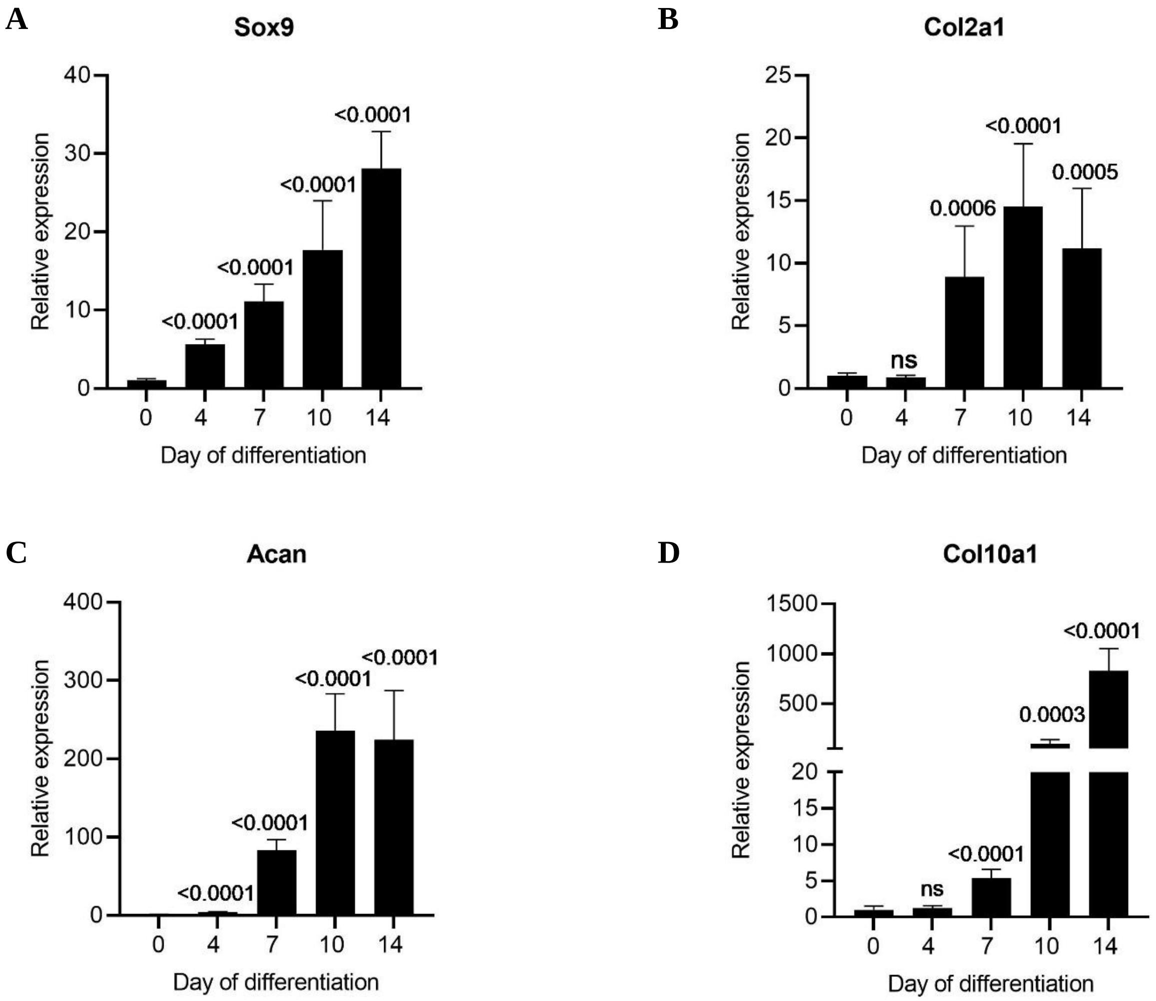

Supplementary Figure 1. Confirmation of chondrogenic differentiation of ATDC5 cells

ATDC5 cells were differentiated in the chondrogenic lineage for 0, 4, 7, 10 and 14 days. A-D) Different stages of chondrogenic differentiation were confirmed by measuring gene expression of Sox9, Col2a1, Acan and Col10a1 by RT-qPCR. Gene-expression was normalized to β-actin expression. Data (mean + standard deviation; n=6 biological replicates) is depicted as fold change relative to t=0. For statistical evaluation an independent samples t-test was performed between each consecutive time point. p-values are indicated.

References

1. Narla, A. and B.L. Ebert, *Ribosomopathies: human disorders of ribosome dysfunction.* Blood, 2010. **115**(16): p. 3196-205.
